# Pembrolizumab-Associated Multiorgan Sarcoid-Like Reaction: A Case Report and Review of Literature

**DOI:** 10.1155/carm/9915002

**Published:** 2025-05-20

**Authors:** Majd Enayah, Tameem Al-Aqtash

**Affiliations:** ^1^School of Medicine, The University of Jordan, Amman, Jordan; ^2^Baylor Scott and White Health System, Plano, Texas, USA

**Keywords:** cancer, case report, immune-related adverse events, immunotherapy, sarcoidosis

## Abstract

**Background:** Immune checkpoints are molecules that serve to augment or inhibit the immune response. The treatment landscape for numerous tumors now relies significantly on immune checkpoint inhibitors (ICIs). Pembrolizumab, a subset of ICIs specifically focused on the programmed cell death 1 (PD-1) molecule. By blocking PD-1, these inhibitors enhance the ability of the immune system to fight cancer cells. Although PD-1 inhibitors are critical in cancer treatment, their use can be associated with immune-related adverse events, such as ICI-related sarcoid-like reaction.

**Case Presentation:** This report describes a 49-year-old female patient with stage IIIA breast cancer breast cancer who developed ICI-related sarcoid-like reaction after starting a neoadjuvant chemoimmunotherapy regimen that included Pembrolizumab. After 4 months of ongoing treatment, she started having significant nausea and vomiting. Computed tomography (CT) scans performed during hospitalization revealed multiple pathologically enlarged thoracic lymph nodes, suspicious for disease progression. Initially, the laboratory workup and cultures were unrevealing. However, esophagogastroduodenoscopy and bronchoscopy were performed, showing noncaseating granulomas in both the stomach and thoracic lymph node biopsy samples. The patient was diagnosed with a sarcoidosis-like reaction to Pembrolizumab. Notably, her symptoms rapidly improved upon initiating systemic corticosteroids. Follow-up CT scan showed a significant improvement in lymphadenopathy after discontinuing Pembrolizumab.

**Conclusion:** This case emphasizes the significance of acknowledging sarcoid-like reactions as possible adverse effects of Pembrolizumab. Given the rising utilization of PD-1 inhibitors, it becomes imperative to be mindful of such adverse events. This awareness helps avoid misdiagnosing disease progression and aids in making informed decisions about ongoing treatment with ICIs.

## 1. Introduction

The advancement of cancer immunotherapies, harnessing the immune system to restore antitumor immunity, has transformed the treatment landscape for many cancers. Immune checkpoints, including cytotoxic T-lymphocyte antigen 4 (CTLA-4), programmed cell death 1 (PD-1), and PD ligand 1 (PD-L1) among others, contribute to the enhancement or the inhibition of the immune response [1]. Immunotherapies directed at these immune checkpoint pathways have shown promise for achieving a lasting response and prolonged disease stability in a notable percentage of inoperable, advanced, or recurrent cancers in patients with multiple types of cancer, with favorable tolerability [2]. Immune checkpoint inhibitors (ICIs), including Ipilimumab, Nivolumab, and Pembrolizumab, are being acknowledged as “life-saving” [3]. Immune-related adverse events linked to ICIs include fatigue, pyrexia, chills, infusion reactions, dermatitis, colitis, and pneumonitis, along with endocrine, liver, renal, and ocular toxicities [3]. These agents may also result in the development of granulomas by inducing macrophage and T-cell modulation [4, 5]. Sarcoid-like reaction is a term used to describe the occurrence of granulomatosis in patients who do not meet the criteria of systemic sarcoidosis [6]. Here, we present a patient with breast cancer who developed a sarcoid-like reaction while being treated with Pembrolizumab.

## 2. Case Description

This is the case of a 49-year-old woman who presented with a right breast mass. A mammogram and ultrasound confirmed the presence of a 4.2 cm lesion. Subsequent biopsy results showed grade 3 invasive ductal carcinoma, with a low ER of 3%, and negative PR and HERE2 by IHC and FISH. Ki 67 expression increased by 76%. Genetic testing for germline mutations was negative. Magnetic Resonance Imaging of the breast confirmed right upper quadrant breast mass with an enlarged right axillary lymph node. Staging imaging, including chest, abdomen, and pelvic CT, as well as nuclear bone scan, was negative for metastatic disease or enlarged lymph nodes, except the right axillary lymph node. The patient breast cancer clinical stage IIIA (cT2 cN1 cM0) according to the AJCC eighth edition [7]. Subsequently, the patient began a neoadjuvant chemoimmunotherapy protocol. The tumor had a very low ER expression and was considered as a triple negative breast cancer affecting the treatment decision incorporating Pembrolizumab immunotherapy with Carboplatin and Paclitaxel, followed by Pembrolizumab combined with Doxorubicin and Cyclophosphamide. Her treatment course was complicated with nausea, vomiting, and decreased oral intake 4 months after starting treatment. The symptoms fluctuated but persisted for the next 3 months while on treatment protocol despite adjusting the chemotherapy dose. She required a brief hospital admission, in which she received supportive care. Eventually, the symptoms were briefly controlled to allow her to undergo right breast-conserving surgery with sentinel lymph node biopsy which showed a complete pathological response to therapy.

**Figure 1:**
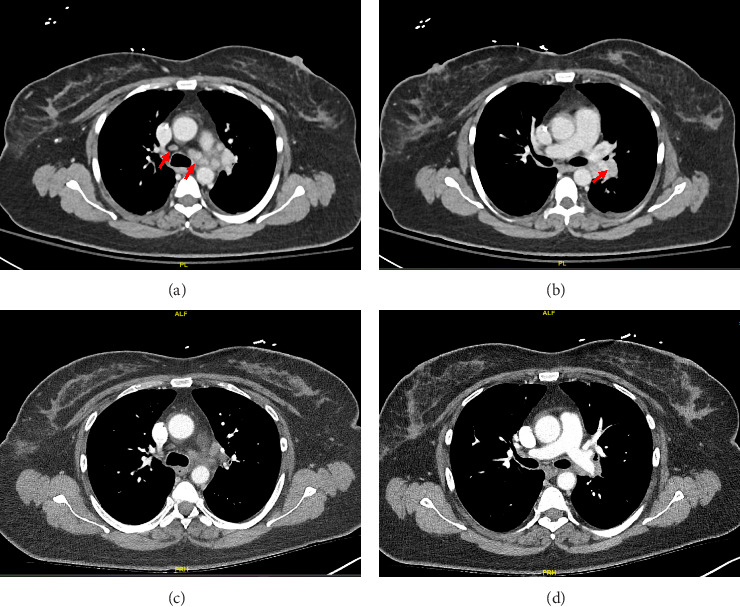
Contrast-enhanced chest computed tomography (CT), contrast-enhanced chest CT scan (a, b) showing enlarged lymph nodes in the mediastinum (a) and left hilum (b) after 8 cycles of neoadjuvant treatment. Contrast-enhanced chest CT scan (c, d) showing improved lymphadenopathy after corticosteroids treatment and 2 months after stopping immunotherapy in the mediastinum (c) and left hilum (d).

Our patient had persistent symptoms of nausea and vomiting. She could not tolerate any oral diet, and she was admitted again to the hospital. The initial hospital assessment revealed severe dehydration and malnutrition, prompting the administration of total parenteral nutrition. A CT scan of her abdomen and pelvis did not show any pathology. Aspiration due to vomiting was suspected, and a chest CT scan was performed, revealing enlarged mediastinal and hilar lymph nodes suspicious for metastatic lymphadenopathy (Figures 1(a) and 1(b)).

**Figure 2:**
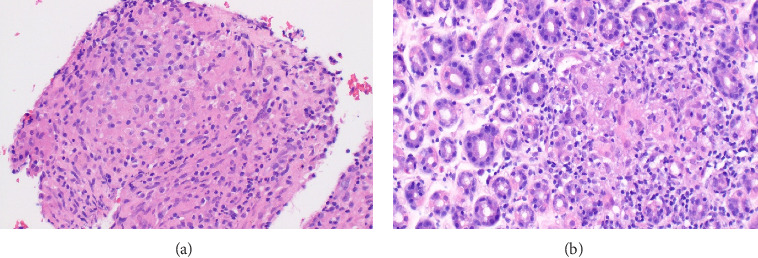
HE stains of the mediastinal lymph node biopsy (a) and gastric biopsy (b) showing noncaseating granulomas, and no tumor cells are present in the biopsy after neoadjuvant therapy.

The patient underwent esophagogastroduodenoscopy that revealed patchy moderate mucosal congestion and granularity in the stomach. Biopsies demonstrated chronic gastritis and noncaseating granulomas. The GMS stain for fungal organisms, the Kinyoun stain for acid-fast bacilli (AFB stain), and the immunostaining for *H. pylori* were all negative. The duodenal biopsy was normal. The patient also underwent bronchoscopy with bronchoalveolar lavage (BAL) and endobronchial ultrasound-guided transbronchial needle aspiration (EBUS-TBNA) of enlarged lymph nodes. The airway exam showed normal mucosa and no endobronchial lesions. BAL of the left upper lobe had mild mixed inflammatory cells. The lavage sample cytology was negative for malignant cells. Biopsies of the left paratracheal lymph node and the left hilar lymph node revealed benign lymphocytes with noncaseating granulomas, no malignant cells were identified (Figure 2). The fungal GMS stain and AFB stain were negative. Laboratory testing showed basic chemistry, liver functions, complete blood count, ANA, ANCA, rheumatoid factor without significant abnormalities. Angiotensin-converting enzyme and calcium levels were normal, as well as 25-vitamin D level.

In this case, we have considered the temporal relationship between Pembrolizumab therapy and the onset of symptoms into our clinical evaluation. Based on laboratory tests, imaging studies and histopathological examination we excluded other etiologies like granulomatous infections and autoimmune disease. The manifestations also did not fit clinical criteria of systemic sarcoidosis. We concluded that patient's symptoms were induced by immunotherapy (Pembrolizumab). Subsequent treatment with Pembrolizumab was discontinued and the patient was started on a steroid regime. Initially, steroids were administered intravenously until the patient was able to tolerate oral intake, followed by 40 mg of prednisone daily tapered over 6 weeks. Two months later, her symptoms had resolved, and a follow-up chest CT scan demonstrated significant improvement in left mediastinal and hilar lymphadenopathy (Figures 1(c) and 1(d)).

## 3. Discussion

This case is among many reported cases of sarcoid-like reaction following treatment with PD-1 inhibitor Pembrolizumab as shown in Table 1. The term “sarcoid-like reaction” typically denotes localized reactions, in contrast to the systemic process observed in sarcoidosis. However, systemic involvement affecting organs like the lungs, skin, and kidneys can also occur [13]. The lungs and intrathoracic lymph nodes are predominant sites of involvement [18]. Pembrolizumab has demonstrated efficacy in improving pathological complete response in combination with neoadjuvant chemotherapy in high-risk triple-negative breast cancer patients [19]. During neoadjuvant therapy, which included Pembrolizumab, our patient experienced thoracic lymphadenopathy with granulomas, along with gastric symptoms related to granulomatous gastritis.

Our patient did not have a compatible clinical presentation of sarcoidosis and lacked other systemic clinical or laboratory features that makes sarcoidosis highly or probable [20]. In addition, her initial chest CT scan before starting treatment did not show any lymphadenopathy. Furthermore, her culture results excluded granulomatous infections, and her symptoms improved after a course of systemic steroids and discontinuation of immunotherapy. The evidence suggests that this patient has suffered from ICI-related sarcoid-like reaction. It would be difficult to know whether ICI therapy may worsen an underlying asymptomatic sarcoidosis or otherwise unmask subclinical autoimmune tendencies. The underlying mechanism of immunotherapy-induced sarcoid-like reaction is still not fully understood, but occurrences have been observed in association with various checkpoint inhibitors, including anti-PD1 targeting agents [21, 22]. Studies propose a connection between sarcoidosis and genetic factors, environmental exposure, and apparent dysregulation of the immune system, characterized by an exaggerated immune response of T helper 1 (Th1) [23]. On the other hand, the PD-1 axis carries out its role by triggering homeostatic inhibition of previously activated T cells. This serves as a self-regulatory pathway within the immune system, preventing the occurrence of overly aggressive immune responses [24]. Therefore, blocking checkpoint inhibitors can lead to increased immune response and autoimmunity, manifesting as immune-related adverse events, such as sarcoid-like reaction. Awareness of sarcoid-like reactions as a potential differential diagnosis for new lung lesions during ICIs therapy is crucial to avoid misdiagnosing tumor progression, thereby preventing confusion in patient management and guiding appropriate treatment decisions [25]. Systemic manifestations of sarcoid-like reactions associated with ICI therapy could be obvious, such as skin lesions [26], or it could be more challenging to connect to a single inciting drug, as in the case of our patient who presented with nausea and vomiting. A thorough multidisciplinary assessment is vital, including biopsies for precise diagnosis and thoughtful exploration of potential differential diagnoses. Overall, the development of immune-related adverse events does not appear to negatively impact survival or pose an immediate threat to patients [27], and therefore the decision on the continuation of ICI therapy should be personalized considering factors such as the severity of symptoms, organ dysfunction, clinical disease status, and the patient's tolerance to ICI therapy [8]. Given the general favorable prognosis and mild clinical presentation of ICI-related sarcoid-like reaction, clinicians must carefully assess the advantages of continuing treatment before making a final decision on the course of action [28]. Typically, patients respond positively to corticosteroids [8] and notably, the use of steroids does not appear to diminish the anti-tumor effects of immunotherapy [29].

## 4. Conclusion

Our case contributes to the expanding literature connecting ICI therapy with rare immune-related adverse events, including sarcoid-like reactions [26, 30–32]. While the specific mechanisms linking PD-1 to sarcoid-like reaction are not yet fully understood, the growing number of reported cases underscores its general involvement in the disease process. This emphasizes the need to be vigilant about the range of toxicities associated with PD-1 inhibitors, especially with the increasing use of ICI in cancer treatment. It raises awareness that PD-1 inhibition, while beneficial in reactivating the immune response to target cancer cells, may inadvertently trigger immune-related adverse events including sarcoid-like reaction.

## Figures and Tables

**Table 1:** Case reports of systemic sarcoid-like reactions in patients treated with Pembrolizumab.

References	Age, sex	Underlying malignancy	Sarcoid organ involvement	Onset	Symptoms	Sarcoid treatment	Outcome
Sirgi et al. [8]	69, F	Non–small cell lung cancer, stage IV	Cutaneous	20 (months)	Nostril lesions	Shave removal of the nostril lesions and received triamcinolone 0.1% topical ointment	Complete resolution of the nostril lesions
			Pulmonary	23 (months)	None	Treatment was not initiated	Continued stable disease on surveillance scans

Lu [9]	80, M	Metastatic melanoma	Mediastinal and bilateral hilar lymphadenopathy	17 (months)	None	Pembrolizumab discontinued Observation	Resolved
			Cutaneous nodules, subcutaneous and deep soft tissue lesions with extensive involvement of bilateral posterolateral chest wall and forearms, and a large soft tissue lesion in the right knee	20 (months)			

Van Willigen et al. [10]	50, F	Metastatic melanoma	Mediastinal and hilar lymph nodes	4 (months)	None	Pembrolizumab discontinued	Resolved
			Pancreas (tail)	6 (months)			
			Paraclavicular, paraesophageal, and para-aortic lymph nodes	1 (year)			
			Thoracic vertebra 7 and the left ileum	15 (months)			

Jespersen et al. [11]	57, F	Metastatic melanoma	Cutaneous	7 (months)	Skin lesion at the elbow	Pembrolizumab was paused and low-dose prednisone was administered (10 mg/day for 2 weeks then 5 mg/day for 2 weeks, then 2·5 mg/day for an additional 2 weeks)	Complete remission Myalgia in her neck and thighs, returned 1 month later and therefore she was again put on a low dose of prednisone (5 mg daily)
			Mediastinal and skeletal (hip bone)	1 (year)	Myalgia in her neck and thighs		

Ahsan et al. [12]	68, M	Invasive melanoma	Mediastinal and hilar lymph nodes	2 (weeks)	None	40 mg prednisone daily for 6 weeks and discontinuation of Pembrolizumab	Improved

Shrateh et al. [13]	69, M	Clear cell renal cell carcinoma	Multiple bilateral subpleural lung nodules	1 (year)	Cough	Symptomatic treatment for cough	Improved
			Mediastinal and hilar lymph nodes				

Lee et al. [14]	50, M	Stage III renal cell carcinoma	Mediastinal and hilar lymph nodes	1 (year)	Cough, fatigue, pruritus, decreased appetite and weight loss	Four weeks prednisone tapered at 40 mg daily and reduced by 10 mg per week	Patient reported a tremendous improvement in energy and appetite by the second week of the prednisone taper. A follow-up chest CT scan 6 weeks after completion of the steroid taper revealed no adenopathy in the chest by CT size criteria

Keane et al. [15]	26, M	Metastatic colon cancer	Intra-abdominal lymph nodes: increased in size New lesion: Left apical consolidation	8 (weeks)	None	None	Radiological evidence of both pulmonary and extra-pulmonary SLR, however he remains clinically well and devoid of any respiratory or other symptoms, and has not required initiation of immunosuppressive therapy for the SLR
			Left upper lobe consolidation: Worsened New lesions: • New and increasing pulmonary nodules • Increased mediastinal and bilateral hilar lymph nodes	28 (weeks)			

Yousuf et al. [16]	62, F	Lung adenocarcinoma	Cutaneous and subcutaneous	9 (weeks)	Right wrist pain and hyperpigmentation of previous scars as well as new skin lesion on her face and subcutaneous thickening of right forearm	Pembrolizumab discontinued and 30 mg prednisone daily tapered over 6 weeks	Marked improvement in her skin lesions and, repeat CT scan after 8 weeks showed resolution ground glass appearance
			Pulmonary		CT thorax showing ground glass changes		

Murgia et al. [17]	55, F	Nonmelanoma skin cancer, specifically facial actinic keratosis	Pulmonary	5 (months)	None	Pembrolizumab discontinued and prednisone at 1 mg/kg	Improved
			Cutaneous		Subcutaneous nodules	Subcutaneous nodules excised from the upper left arm and lower left leg	

This case	49, F	Stage IIIA triple negative breast cancer	Mediastinal and hilar lymph nodes	4(months)	None	Steroids were administered intravenously until the patient was able to tolerate oral intake, followed by 40 mg of prednisone daily tapered over 6 weeks	Two months later, all her symptoms resolved, and a follow-up chest CT scan demonstrated significant improvement in left mediastinal hilar lymphadenopathy
			Gastric		Nausea, vomiting and deceased oral intake		

## Data Availability

Data sharing is not applicable to this article as no datasets were generated or analyzed during the current study.
